# Impact of feature saliency on visual category learning

**DOI:** 10.3389/fpsyg.2015.00451

**Published:** 2015-04-21

**Authors:** Rubi Hammer

**Affiliations:** Department of Communication Sciences and Disorders, Interdepartmental Neuroscience Program, Northwestern UniversityEvanston, IL, USA

**Keywords:** category learning, feature saliency, supervised learning, visual attention, visual perception, visual expertise, unsupervised learning

## Abstract

People have to sort numerous objects into a large number of meaningful categories while operating in varying contexts. This requires identifying the visual features that best predict the ‘essence’ of objects (e.g., edibility), rather than categorizing objects based on the most salient features in a given context. To gain this capacity, visual category learning (VCL) relies on multiple cognitive processes. These may include unsupervised statistical learning, that requires observing multiple objects for learning the statistics of their features. Other learning processes enable incorporating different sources of supervisory information, alongside the visual features of the categorized objects, from which the categorical relations between few objects can be deduced. These deductions enable inferring that objects from the same category may differ from one another in some high-saliency feature dimensions, whereas lower-saliency feature dimensions can best differentiate objects from distinct categories. Here I illustrate how feature saliency affects VCL, by also discussing kinds of supervisory information enabling reflective categorization. Arguably, principles debated here are often being ignored in categorization studies.

Starting at infancy, we skillfully categorize visually perceived objects in every conscious moment of our lives. However, much is still unknown about the underlying cognitive mechanisms of visual category learning (VCL). If asking a young child how she can tell apart dogs from cats, she would probably say something like “dogs look like other dogs, but they do not look like cats,” possibly being surprised at being asked such a question. Undeniably, when lacking any specific knowledge, categorizing objects based on their overall perceived similarities seem to be reasonable. However, most often categorizing based on overall similarities is maladaptive; and possibly even at early development, categorization is affected by acquired attentional biases or by ‘inherent core-knowledge’ (Spelke and Kinzler, 2007; Pereira and Smith, 2009).

Understanding the challenges in VCL requires characterizing the sensory input and the minimal processes enabling production of a satisfactory categorization decision (output). VCL essentially involves identifying perceived features (e.g., shape or color) that predict important characteristic of an object (e.g., edibility). People act, develop, and evolve in a cluttered and ever-changing environment, where often there is a mismatch between the environmental objective structure, and the subjective interpretation of the environment required for adaptive behavior. Under such conditions, irrelevant visible features may initially be perceived as most salient and thus they may overshadow less salient and possibly more important features. Consequently, VCL requires resolving two primary challenges: (i) Learning to ignore salient irrelevant variability. (ii) Identifying and becoming more sensitive to important between-categories differences, even if these are not salient. Ultimately, the acquired category representation has to be robust, so as to be applicable in different contexts.

Here I exemplify how feature saliency affects categorization in different scenarios, and what is required for categorization not to be exclusively driven by feature saliency. While principles debated here were previously discussed, separately, currently there is no coherent overview of the topic. Moreover, interactive effects between feature saliency and supervisory information that is made available to subjects in VCL studies are too often underestimated or overlooked. Evidently, properly accounting to such effects is essential for dissociating experimental results reflecting objective (contextual) characteristics of VCL tasks, from those characterizing human cognition.

## Impact of Respective Feature Saliency on VCL

**FIGURE 1:**
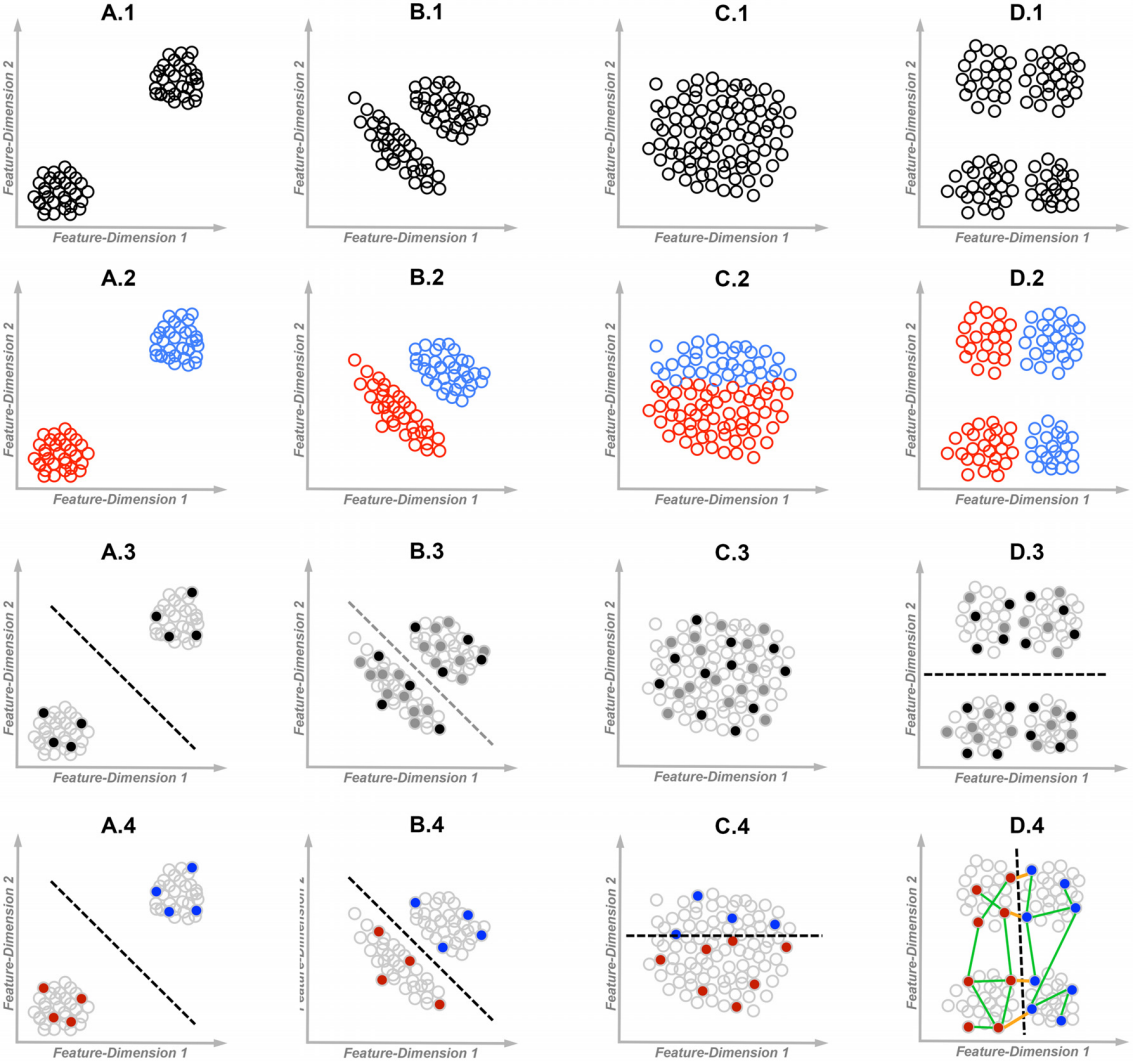
**Examples of different visual category learning (VCL) scenarios from the least dependent **(A)** to the most dependent **(D)** on supervision.** Each dot represents an exemplar, where exemplars may differ in two visually perceived feature dimensions. Dashed lines represent the likely to be deduced category decision boundaries. **Row-1**: The unlabeled data sets; **Row-2**: The fully color-labeled data sets; **Row-3**: Filled dots represent the observed exemplars available for unsupervised statistical learning (black-dots represent a likely to be ‘too sparse’ observed sample in B3). Gray-circled empty dots are the unobserved exemplars; **Row-4**: Filled dots represent color labeled exemplars. Green lines (D4) represent some positive equivalence constraints or ‘must be related’ paired exemplars, whereas orange lines represent negative equivalence constraints or ‘cannot be related’ paired exemplars.

In some scenarios, categorization can be driven exclusively by objects’ visual characteristics, and therefore it does not necessitate learning and may seem ‘reflexive’ (**Figure 1A**), or it can be resolved by learning the statistics of visual features by observing multiple objects (**Figure 1B**). In other scenarios, supervised learning is essential for identifying which visual features are important for categorization and which are not (**Figures 1C,D**). Supervised learning can often be self-governing and accomplished without the guidance of an ‘expert tutor’; but in some scenarios, VCL can be effective only if an effort is invested in structuring the learning environment. This later form of learning is perhaps unique to humans, characterizing (but not restricted to) educational settings and scientific explorations. The four VCL scenarios illustrated in **Figure 1** operationally differ in the respective saliency of within-category differences versus between-categories differences. Correct categorization in the scenario illustrated in **Figure 1A** requires minimal supervision, whereas the one illustrated in **Figure 1D** requires the most effortful supervision. Being able to adapt to all these scenarios allows us to become reflective decision makers, capable of altering the environment according to our needs, instead of being ‘reflexive’ creatures, driven by few most salient perceptual characteristics of the environment.

The simplest organisms capable of ‘acting’ based on visual information have no capacity to learn. Some single-celled organisms are capable of swimming toward (or away from) a light source following the steepest light gradient (phototaxis), migrating to an environment that better fit their needs. Such apparently simplistic yet adaptive ‘categorical decisions’ are also part of human experience. For example, infants can differentiate between dogs and cars (but not between dogs and rabbits) without any prior guidance (Quinn and Johnson, 2000). This example involves two homogeneous basic-level categories, each associated with a distinct superordinate-level category (Rosch and Mervis, 1975). Here, each two exemplars from within a given basic-level category differ, at most, in very few salient features, whereas two exemplars from the contrasted superordinate categories differ in multiple salient features (**Figure 1A**), and thus ‘reflexive categorization’ is feasible (Macé et al., 2005). In such scenarios it may even seem as if people can ‘categorize’ objects as fast as they can detect the presence of an object (Grill-Spector and Kanwisher, 2005). However, in studies with categories compositions as described in **Figure 1A**, actions such as gaze-duration, eye-saccades, key-pressing, or category-specific neural activity, *do not necessarily* reflect *acquired* knowledge or the capacity to generalize from past experiences following VCL. Such studies teach us little about neurocognitive mechanisms of knowledge-based categorization (though they may teach us about lower-level visual processing). These should not be confused with studies in which participants perform VCL tasks where differences between objects from a given category are as salient as differences between objects from the contrasted categories, and where the lower-level characteristics of stimuli in the contrasted categories are largely matched (e.g., Folstein et al., 2013; Hammer et al., 2015).

Evidently, improper selection of stimuli may result in confusion between bottom-up effects driven by feature saliency and knowledge-based effects. For example, showing that the human lateral occipital cortex (LOC) is most sensitive to differences between dogs and flowers (or between airplanes and shoes) does not indicate that the LOC is most sensitive to taxonomic basic-level categories, or any other form of object-level acquired knowledge, as suggested by Iordan et al. (2015). Basic-level is considered as the categorization level that is most *culturally* salient, implying an acquired organization of categories with *subjective* importance, rather than organization formed solely by *objective* feature saliency (Rosch and Mervis, 1975). Essentially, contrasting dogs with flowers, or shoes with airplanes, is simply contrasting between two relatively homogeneous sets of stimuli that differ from one another in multiple salient features (and it is comparable, for example, to contrasting dogs with airplanes). Properly testing sensitivity to basic-level categories should involve, for example, testing sensitivity to differences between rabbits and dogs, cars and trucks, or fruits and vegetables (distinct, taxonomically-meaningful categories that share much of their low-level perceptual properties). Given the categories they contrasted, the findings reported by Iordan et al. (2015; see also Cichy et al., 2014) may only reflect a generic LOC sensitivity to differences in intermediate-level features (e.g., prevalence of curved edges or large monochromatic blobs), rather than sensitivity to basic-level categories (Op de Beeck et al., 2008; Gilbert and Li, 2012).

Unlike the scenario described in **Figure 1A**, where correct categorization can be accomplished by observing few exemplars, unsupervised VCL often requires sampling a large number of exemplars before correct generalization becomes possible (Fried and Holyoak, 1984; Rosenthal et al., 2001; Kloos and Sloutsky, 2008; Turk-Browne et al., 2008). In the scenario illustrated in **Figure 1B**, each exemplar has less salient differences from nearby exemplars within its own category, as compared with the nearest exemplars from the other category, enabling unsupervised VCL (Jain, 2010). Nevertheless, here VCL requires sampling multiple exemplars that correctly represent the distribution/densities of objects’ features. For example, being introduced with all the filled exemplars in **Figure 1B3** (without knowing any category labels) is sufficient for learning the densities and the categories’ boundaries. A too sparse sampling of exemplars (e.g., sampling only the black filled exemplars) would result in more salient differences within the sample of each category, an errorful learned representation of categories, and a greater chance for later categorization errors. This is likely to result in ineffective unsupervised VCL, specifically when within-category differences are as salient as between-categories differences (Ell and Ashby, 2012). On the other hand, a preselected biased sample of exemplars that hints about a between-categories boundary, such as multiple successive trials presenting exemplars from the same category, is likely to facilitate ‘unsupervised’ VCL (Zeithamova and Maddox, 2009; Gershman and Niv, 2013; Clapper, 2014). I suggest that VCL tasks that do not involve the use of labels or feedback, yet involve a biased selection of exemplars, should be considered as supervised tasks – in effect, a biased sampling of exemplars provides participants with implicit supervisory information by revealing part of the experimenter knowledge of the categories structure (see Palmeri and Mack, 2015 for a related discussion).

In scenarios such as those illustrated in **Figures 1C,D**, it is impossible to infer the underlying categories structure from the distributions of objects’ features. Specifically, while in **Figure 1C** there is no structure that can be discovered by unsupervised mapping of densities, in **Figure 1D** mapping the densities may be misleading (see **Figure 1D3** for the likely inferred decision boundary). Clearly, here additional information is required in order to uncover underlying patterns with potential significance. Such information may include labeled exemplars or an intentionally biased sample of exemplars, selected by an ‘expert tutor.’ For example, being introduced with the labeled (filled) exemplars in **Figures 1C4,D4** is sufficient for learning the categories’ boundaries. Labeled exemplars are with little or no use for VCL in a scenario such as illustrated in **Figure 1A**, but they may facilitate VCL in a scenario such as illustrated in **Figure 1B** (specifically when the sampled exemplars are too few and thus the categories’ boundaries become fuzzy, e.g., as when observing only the black filled exemplars in **Figure 1B3**).

## Supervision Allows Categorization to be Less Affected by Feature Saliency

Unsupervised VCL is likely to be effective if an objective category structure is consistent with the subjective needs of the learning organism. As much as this may satisfy the needs of simple organisms acting in a largely fixed environment, unsupervised VCL is unlikely to satisfy the needs of organisms acting in different environments where multiple expertise are required for survival. In supervised VCL, in addition to the visually perceived features of the categorized objects (target sensory input), supervisory information that enables inferring an important relation between objects, and sometimes also the essence of objects, is also available. Supervisory information can become available to the learning organism following earlier categorization decisions it has made, or independently of its actions. In real-life scenarios, VCL is likely to be semi-supervised, where the supervisory information for only a small sample of objects is available.

Supervised VCL can be based on an operant conditioning processing pipeline that includes perceiving a target object, followed by executing a specific action that reflect the organism’s initial hypothesis regarding the nature of the perceived object. This action may trigger new sensory impressions (primarily a reward or punishment), which can be used as feedback indicating the correctness of the initial hypothesis. For example, operant conditioning allows bees to learn that approaching a virtual blue flower (regardless of its spatial location) results in receiving a reward (sugar solution), whereas approaching blue–green flowers results in an aversive experience (receiving quinine solution). This indicates that attention control and motivational processes impact supervised VCL, even in simple organisms such as bees (Giurfa, 2013). Supervisory information available in operant conditioning enables ‘deeper’ realizations (e.g., “blue flowers are good,” whereas “blue–green flowers are bad”).

Supervised VCL can also be based on classical conditioning, where exemplars that share visual features (conditioned stimuli) are consistently associated with a specific rewarding or aversive experience (unconditioned stimulus). Unlike operant conditioning, classical conditioning is more passive, where the learning organism is introduced with the paired stimuli (unconditioned and conditioned) regardless of its actions. For example, one can autonomously learn that when dark-gray clouds are forming in the sky (conditioned stimulus) soon it would rain heavily (a specific unconditioned aversive stimulus), whereas light-gray clouds do not pose any threat. Similarly, a mother walking with here child in the park while referring to several animals by pointing at them and saying “look! a dog!” allows the childe to learn that all these animals are of the same kind. This may allow the child to learn to ignore irrelevant salient perceived differences between dogs, such as fur texture and body size, and to acquire a generalized representation of dogs. In this latter example, instead of an inherently rewarding or aversive unconditioned stimulus, a category-label (“dog”) was used. Here the toddler first has to learn that a label is a proxy – it symbolizes a meaningful category of objects or events. This requires an intermediate learning phase where, as part of the process of language acquisition, the toddler learns about the importance of labels (see Graham et al., 2012; Robinson and Best, 2012; Sloutsky and Fisher, 2012, for a related debate).

Essentially, all kinds of supervisory information allow identifying relevant features by imposing equivalence constraints – informing the learning organism that few example objects are likely to be from the same-category or from different-categories (Hammer et al., 2008). Unlike unsupervised learning, if the compared or contrasted objects are properly chosen, this may enable effective VCL of a complex categorization rule by using very few constrained training examples (see **Figure 1D4** for the “must be related” and “cannot be related” exemplars). Specifically, comparing two objects while being cued that the two are from the same category is most effective for identifying unimportant salient differences between objects from the same category. As the number of salient differences between the compared same-category exemplars is larger, more information is gained, since more is learned about the permitted variability within a category. On the other hand, contrasting two objects from two distinct categories is most effective for detecting important low-saliency differences between the two categories. This would be effective mostly when the contrasted exemplars differ in very few important features (Hammer et al., 2009a,b, 2010).

Visual category learning that is based on directly being informed about the categorical relation between few objects is sufficient for learning which features are most relevant for categorization, for learning to overlook salient irrelevant within category variability in a given feature dimension, or for becoming sensitive to important low-saliency differences between-categories. However, it may not be sufficient for learning the essence of categories. For example, learning that brown spotted mushrooms are of a distinct kind helps in correctly categorizing mushrooms; but it is insufficient for determining mushrooms’ edibility. On the other hand, supervised VCL with feedback (eating mushrooms) or labels (being told “edible” or “poisonous”) enables both inferring categorical relations between objects, and associating categories with meaningful events such as a rewarding experience, an aversive experience, or a meaningful symbolic representation.

## Biases in Respective Feature Saliency may Serve as ‘Implicit Supervision’

If lacking prior domain-specific knowledge, and if supervision is not available, categorization would rely on the most salient differences between objects (test your initial impression in **Figure 1D1**). However, this principle is often ignored in studies investigating children conceptual knowledge by using visual stimuli or physical objects. For example, according to the shape bias hypothesis, young children (but not adults) systematically generalize object names based on objects’ overall shape (Landau et al., 1988; Hahn and Cantrell, 2012; Yee et al., 2012). In contrast, others showed that some contextual factors eliminate children’s preference to shape (Cimpian and Markman, 2005; Tek et al., 2012; Perry et al., 2014). Most relevant to the current discussion, Hammer and Diesendruck (2005; see also Diesendruck et al., 2003) systematically tested how the respective saliency of computer-animated objects’ shapes versus the saliency of their animated functions, affects object naming in preschool children and adults. In a higher-shape-saliency condition, differences in shapes were more salient than differences in functions. In a higher-function-saliency condition, differences in functional features were more salient than differences in shapes (stimuli examples: https://sites.google.com/site/rubihammer/other-stuff/stimuliexamples). While adults consistently categorized objects based on functional similarities, regardless of the respective saliency of features, children were likely to consider objects as having the same name if they were similar in the feature in which differences were most salient in a given context (either shape or function). Moreover, children’s object naming in a later categorization task, where the saliency of shapes and functions were matched, was biased such that they extended names based on the feature that was with a greater saliency in the earlier task. That is, an early *objective* bias in feature saliency resulted in a *learned* bias that affected later behavior.

The above shows that contextual biases in respective feature saliency may act as ‘implicit supervision,’ guiding (or misguiding) participants. Overlooking such experimental effects may hinder the studying of *intrinsic* feature preferences. I do not claim that early-developed attentional biases do not exist; but I do suggest that in order to properly determine the soundness and robustness of a hypothesized feature preference or an *intrinsic* attentional bias, it is necessary to test subjects in several scenarios where the respective saliency of within-category and between-categories differences is systematically manipulated. This would reduce the odds that experimental findings would be altered by an accidental bias in respective feature saliency, and may enable to better study human cognition. Although the impact of contextual factors on shape bias in children was previously discussed (Samuelson and Bloom, 2008), respective feature saliency is particularly important since it may compromise the studying of top–down knowledge-based processes in most experimental settings (yet it is relatively straightforward administrating tasks in which feature saliency is controlled).

## Usability of Explicit Supervisory Information Relies on Absolute Feature Saliency

Due to initial poor representation in visual cortices, important low-saliency differences between objects may be left undetected even at the absence of higher-saliency distractors. While sensitivity to low-saliency differences can be increased via perceptual learning, for such an improvement to be effective a thoughtful selection of the training examples is required. Specifically, low-saliency scenarios require presenting the learner with exemplars from the two contrasted categories, which differ only in one important feature dimension at a time. Thus, it is expected that in VCL tasks, where categories differ in multiple low-saliency features, learning would be significantly impaired if supervision does not involve an intentional selection of the training examples. On the other hand, VCL in analogs tasks, where categories differ in higher-saliency features, may be less affected by an arbitrary selection of the training examples (Hammer et al., 2008, 2012, 2015). A VCL study, or real-life learning session, administered without accounting for these facts, is likely to yield different results from a VCL session in which an effort was invested in the design and administration of the training trials.

## Conflict of Interest Statement

The author declares that the research was conducted in the absence of any commercial or financial relationships that could be construed as a potential conflict of interest.
